# Fibrothecoma with pseudo-carcinomatosis in a young woman: The radiologic pitfalls of Meigs syndrome and the importance of fertility-sparing surgery

**DOI:** 10.1016/j.radcr.2026.07.130

**Published:** 2026-08-31

**Authors:** Rihab Amara, Mostafa-Yassine Tahouna, Hiba Kourchi, Nadia Mouna, Tarik Deflaoui, Hamid Ziani, Siham Nasri, Imane Skiker

**Affiliations:** a Department of Radiology, Mohammed VI University Hospital, Oujda, Morocco; b Department of General Surgery, Mohammed VI University Hospital, Oujda, Morocco

**Keywords:** Ovarian fibrothecoma, Meigs syndrome, Ovarian mass, Pseudo-carcinomatosis, Computed tomography, Fertility-sparing surgery, CA-125 elevation

## Abstract

Ovarian fibrothecomas are uncommon benign sex cord-stromal tumors that may rarely present with ascites and pleural effusion, constituting Meigs syndrome. In young women, this presentation may mimic advanced ovarian malignancy with peritoneal carcinomatosis, potentially leading to unnecessarily aggressive surgery and avoidable loss of fertility. We report the case of a 38-year-old nulliparous woman presenting with progressive abdominal distension. Contrast-enhanced computed tomography (CT) demonstrated a giant right ovarian mass measuring 198 × 179 mm associated with abundant ascites and left pleural effusion. Multiplanar reconstructions revealed a solid ovarian lesion with suspicious peritoneal nodularity initially interpreted as carcinomatosis. Serum CA-125 level was elevated (275 U/mL), further increasing suspicion for ovarian malignancy. Surgical exploration demonstrated a large right ovarian mass without diffuse peritoneal carcinomatosis. The contralateral adnexa appeared normal. Right adnexectomy with omental and peritoneal biopsies was performed as a fertility-preserving strategy. Cytologic analysis of ascitic fluid showed hemorrhagic inflammatory fluid without malignant cells. Histopathological examination confirmed ovarian fibrothecoma without evidence of malignancy. This case highlights a major diagnostic pitfall in gynecologic imaging: benign ovarian fibrothecoma associated with Meigs syndrome may closely simulate advanced ovarian cancer, particularly in the presence of ascites, pleural effusion, elevated CA-125, and suspicious peritoneal nodules. Increased radiologic awareness of this entity is essential in young women to avoid unnecessarily radical surgery and preserve reproductive potential.

## Introduction

Ovarian fibrothecomas are benign sex cord-stromal tumors composed of varying proportions of fibrous tissue and theca cells. They account for approximately 1%-4% of all ovarian neoplasms and predominantly occur in peri- or postmenopausal women [1,2]. Although generally asymptomatic and benign, large fibrothecomas may be associated with ascites and pleural effusion, forming the classic triad of Meigs syndrome [3].

The association of a benign ovarian tumor with pleural effusion, abundant ascites, elevated CA-125 levels, and suspicious peritoneal abnormalities constitutes a major diagnostic challenge because it may strongly mimic advanced epithelial ovarian carcinoma with peritoneal carcinomatosis [4,5]. This pitfall is particularly critical in young women, in whom misdiagnosis may result in unnecessarily extensive cytoreductive surgery and irreversible impairment of fertility.

Cross-sectional imaging plays a central role in preoperative assessment. However, imaging findings are often nonspecific. Fibrothecomas may appear as large heterogeneous solid adnexal masses with delayed enhancement, degeneration, edema, or cystic changes, potentially simulating malignant ovarian neoplasms [6,7]. The presence of ascites and pleural effusion further increases diagnostic uncertainty.

We report the case of a giant ovarian fibrothecoma associated with Meigs syndrome in a 38-year-old nulliparous woman initially suspected of having ovarian malignancy with peritoneal carcinomatosis. This case emphasizes the importance of careful radiologic interpretation and conservative surgical management in young patients.

## Case presentation

A 38-year-old nulliparous woman presented with progressive abdominal distension evolving over 2 months, without associated digestive, urinary, or gynecologic symptoms. Her medical history was notable for medically treated undocumented psychosis, with no prior surgical history. The clinical course evolved in an afebrile context with preserved general condition. On admission, the patient was conscious, hemodynamically stable, and had an Eastern Cooperative Oncology Group performance status of 0. Physical examination revealed a large abdominopelvic mass extending from the right iliac fossa to the epigastric region, measuring approximately 20 × 15 cm. The abdomen was soft, without signs of peritoneal irritation. Laboratory investigations demonstrated mild anemia (hemoglobin: 10 g/dL) without inflammatory syndrome, while serum CA-125 level was elevated at 275 U/mL.

### Imaging findings

**Fig. 1:**
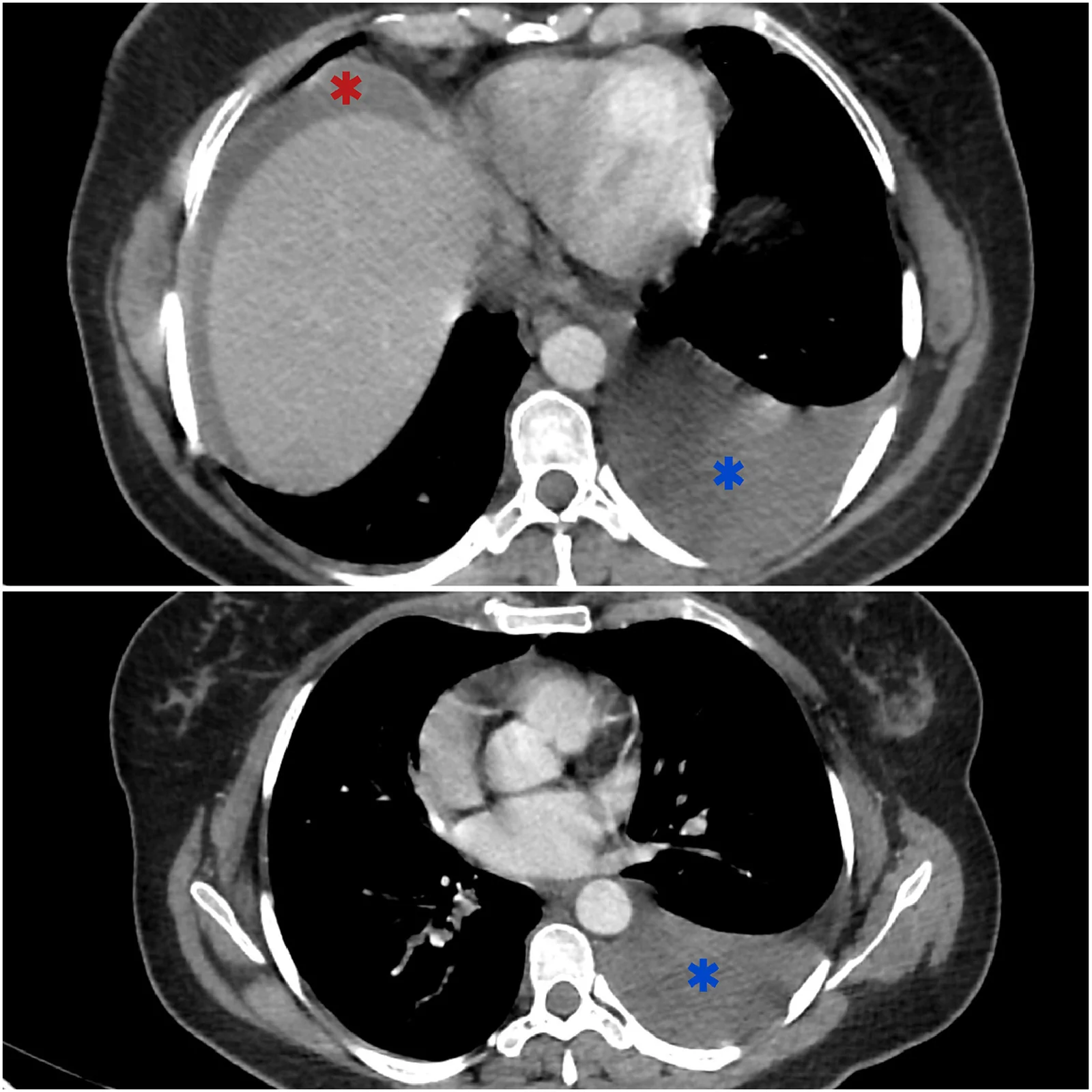
Axial contrast-enhanced CT images showing abundant intraperitoneal ascites (red asterisk) associated with left pleural effusion (bleu asterisk) in the setting of a giant right ovarian mass.

**Fig. 2:**
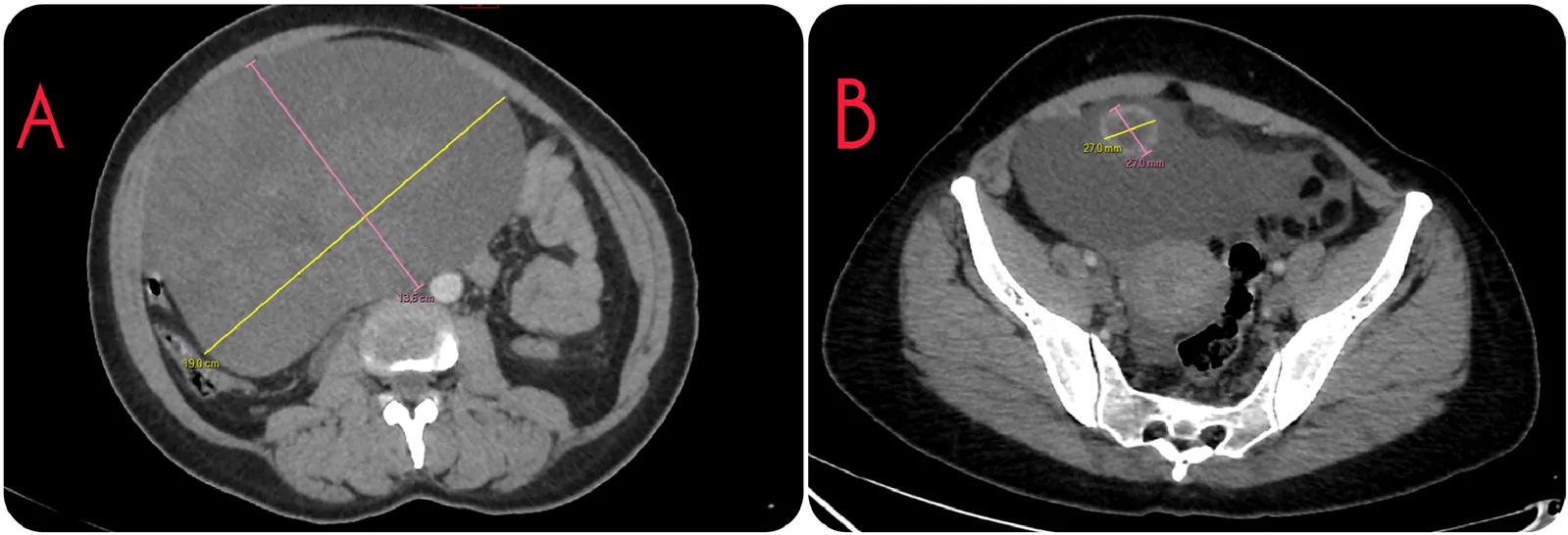
Axial contrast-enhanced CT images demonstrating the superior (A) and inferior (B) portions of the giant right ovarian mass. A nodular peritoneal lesion initially suspected for carcinomatosis is visible adjacent to the inferior aspect of the tumor.

**Fig. 3:**
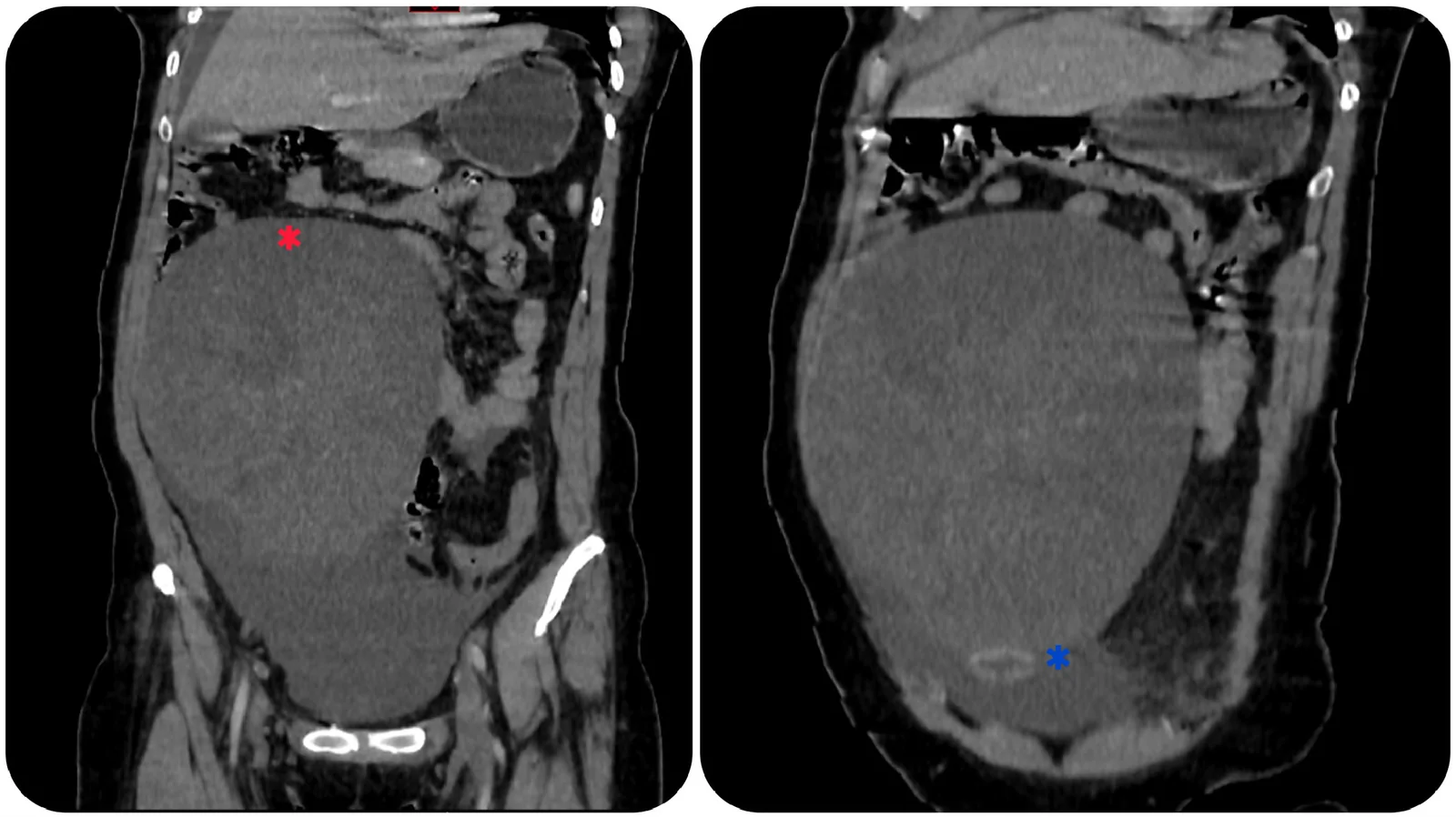
Coronal contrast-enhanced CT reconstructions showing the cranial (red asterisk) and caudal (bleu asterisk) extent of the ovarian mass and its relationship with adjacent abdominal structures.

**Fig. 4:**
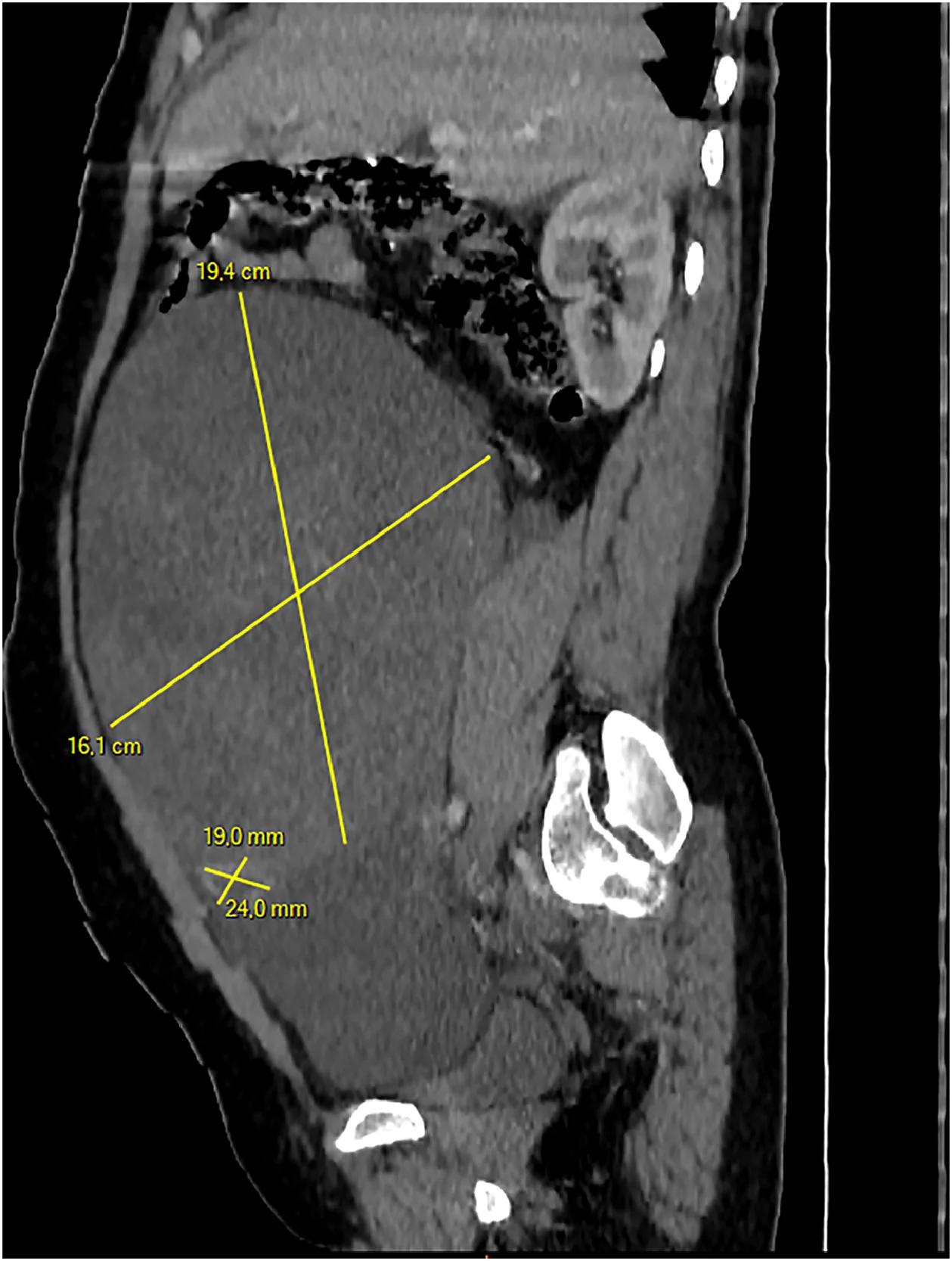
Frontal CT reconstruction demonstrating the giant ovarian mass associated with abundant ascites and inferior nodular peritoneal abnormality.

Contrast-enhanced abdominopelvic CT (Video. 1) demonstrated a giant right ovarian mass measuring 198 × 179 mm associated with abundant intraperitoneal ascites and left pleural effusion (Fig. 1). The lesion appeared predominantly solid with heterogeneous enhancement. Multiplanar reconstructions showed a well-defined adnexal mass occupying a large portion of the abdominopelvic cavity and displacing adjacent digestive structures without clear evidence of direct invasion. A single nodular peritoneal abnormality located near the inferior aspect of the mass was initially considered suspicious for peritoneal carcinomatosis (Fig. 2, Fig. 3, Fig. 4). The association of a giant adnexal mass, ascites, pleural effusion, and elevated CA-125 level strongly suggested advanced ovarian malignancy on initial radiologic interpretation.

However, retrospective analysis demonstrated several elements favoring a benign stromal tumor:

• Relatively smooth tumor margins.
• Absence of infiltrative peritoneal thickening.
• Lack of distant metastatic disease.
• Preserved contralateral adnexa.
• Absence of pathological lymphadenopathy.

The left ovary appeared normal on imaging.

### Differential diagnosis

The principal differential diagnoses included:

1. Epithelial ovarian carcinoma with peritoneal carcinomatosis
  Initially favored because of the association of a large ovarian mass, ascites, pleural effusion, elevated CA-125, and suspicious peritoneal nodules.
2. Ovarian fibroma/fibrothecoma with Meigs syndrome
  Considered retrospectively because of the solid appearance of the mass, smooth contours, and lack of true invasive features.
3. Brenner tumor
  Less likely given the giant size and absence of characteristic calcifications.
4. Pedunculated uterine leiomyoma
  Excluded by identification of ovarian origin and normal uterine morphology.
5. Thecoma or other sex cord-stromal tumors
  Considered due to the predominantly solid stromal appearance.

## Management and outcome

Surgical exploration was performed (Video. 2).

Intraoperatively, a giant right ovarian mass associated with abundant ascites was identified. Careful exploration of the peritoneal cavity demonstrated no evidence of diffuse peritoneal carcinomatosis. The nodular lesion initially suspected on preoperative imaging to represent peritoneal carcinomatosis corresponded intraoperatively to a localized indurated area at the inferior base of the ovarian mass, without macroscopic peritoneal tumor dissemination. The left ovary and fallopian tube appeared macroscopically normal.

Given the young age of the patient, the nulliparous status, the normal appearance of the contralateral adnexa, and the absence of obvious disseminated malignancy, a fertility-preserving surgical approach was adopted. Right adnexectomy associated with omental and peritoneal biopsies was performed.

Cytologic analysis of ascitic fluid demonstrated hemorrhagic inflammatory fluid without malignant cells.

Histopathological examination confirmed ovarian fibrothecoma without evidence of malignancy. Peritoneal and omental biopsies showed no tumor infiltration.

**Fig. 5:**
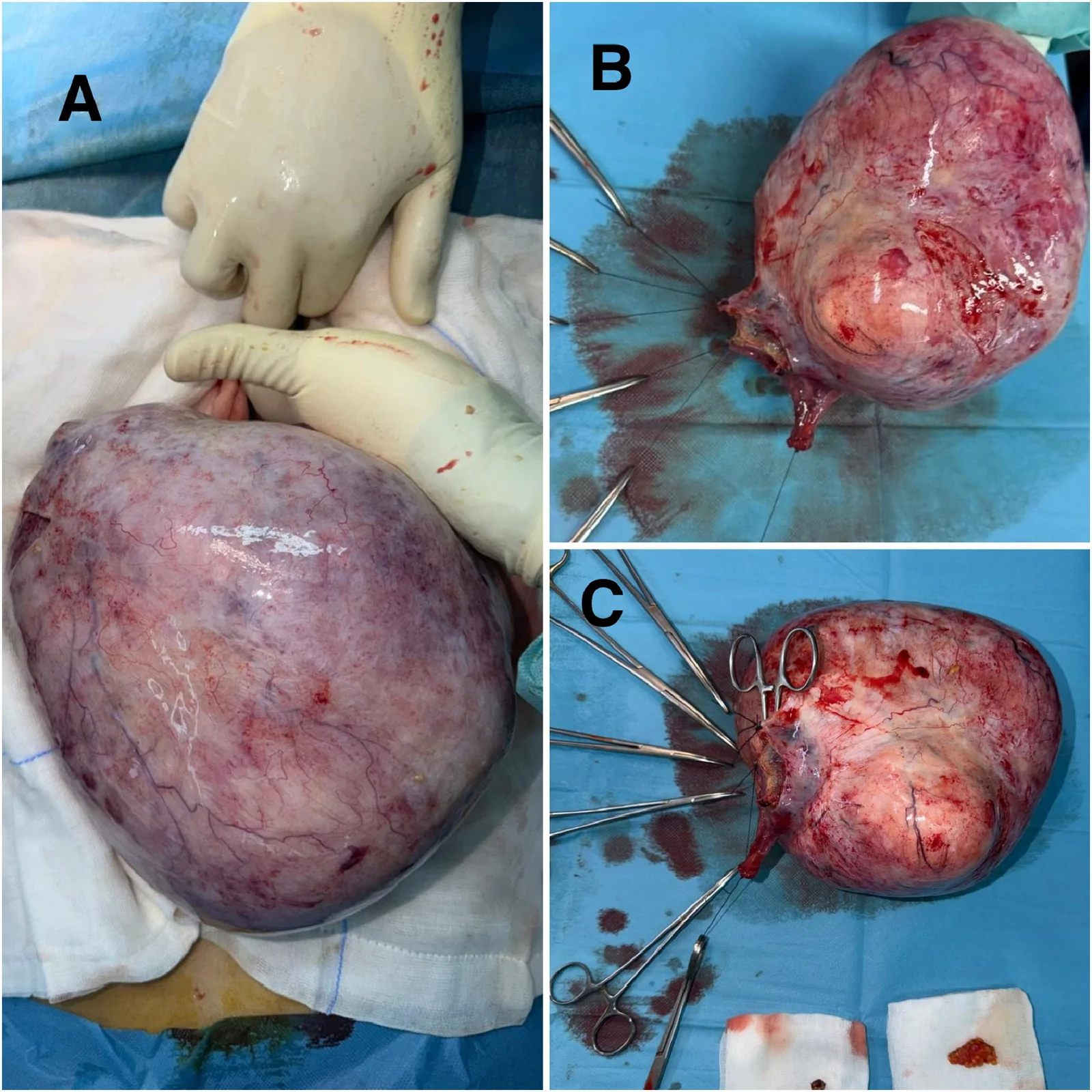
Intraoperative photographs: (A) Giant right ovarian mass before resection. (B) Surgical specimen—anterior surface. (C) Surgical specimen—posterior surface.

**Fig. 6:**
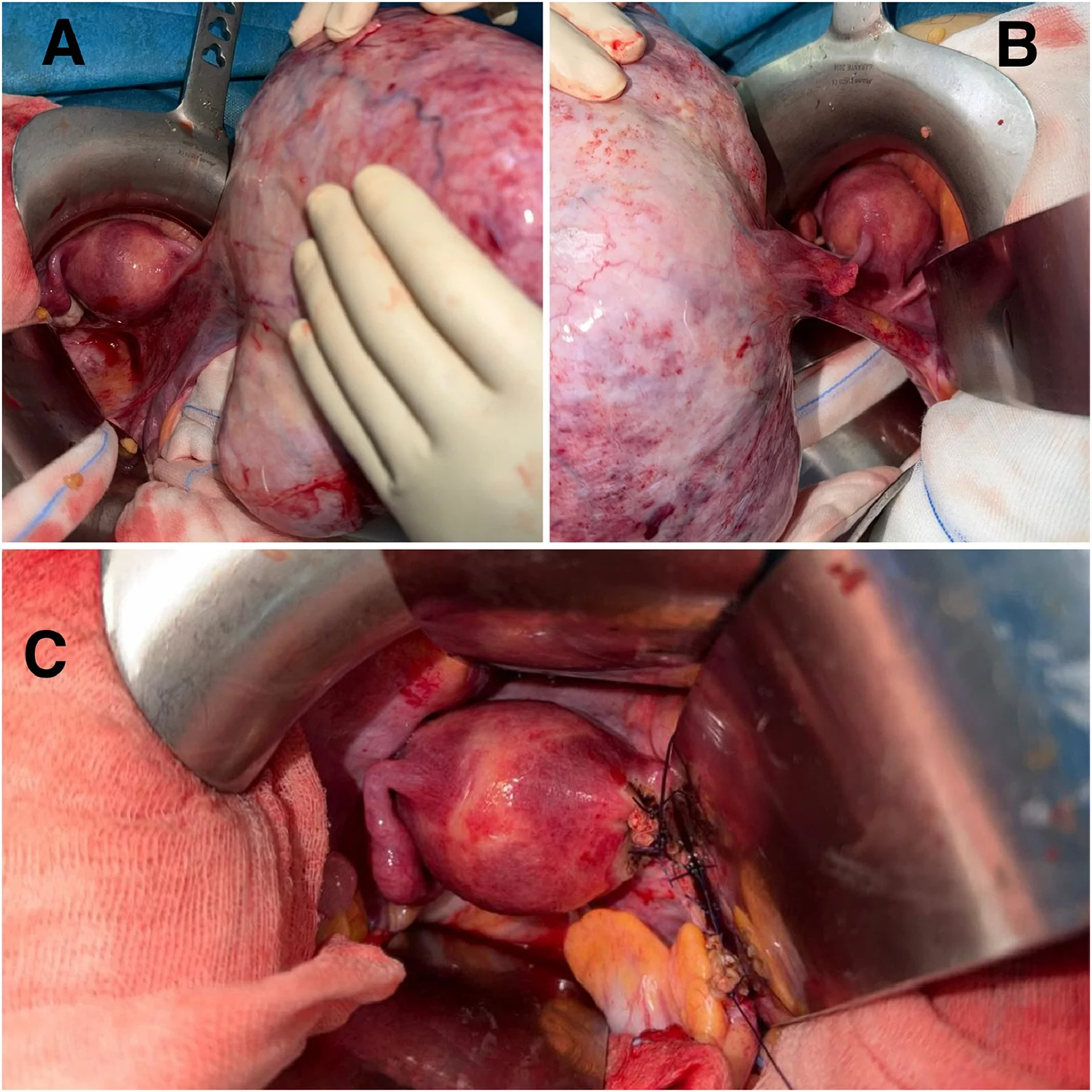
Intraoperative photographs: (A) Normal-appearing left ovary and fallopian tube. (B) Giant right ovarian mass. (C) Sectioned surgical specimen after right adnexectomy.

Postoperative recovery was uneventful, with progressive resolution of ascites and no evidence of recurrence during early follow-up (Fig. 5, Fig. 6).

## Discussion

### Fibrothecoma and Meigs syndrome

Ovarian fibrothecomas belong to the spectrum of ovarian sex cord-stromal tumors and are composed of varying proportions of fibroblastic and theca cells [1]. They are usually benign and unilateral. Large tumors may produce ascites due to transudation of fluid from the tumor surface or mechanical irritation of the peritoneum [8].

Meigs syndrome classically refers to the association of a benign ovarian fibroma or fibrothecoma with ascites and pleural effusion that resolve after tumor resection [3]. Pleural effusion is most commonly right-sided, although left-sided or bilateral involvement may occur [9].

### Mimicry of ovarian carcinoma

The present case illustrates one of the most important pitfalls in gynecologic oncologic imaging: benign fibrothecoma associated with Meigs syndrome may closely mimic advanced ovarian carcinoma.

Several imaging and biologic features contributed to the initial suspicion of malignancy:

• Giant adnexal mass
• Abundant ascites
• Pleural effusion
• Elevated CA-125
• Suspicious peritoneal nodules

CA-125 elevation does not necessarily indicate malignancy in Meigs syndrome and may result from mesothelial irritation caused by ascites and pleural inflammation [10]. Markedly elevated CA-125 levels have been repeatedly reported in benign fibrothecoma-associated Meigs syndrome, leading to frequent diagnostic confusion [11].

Radiologically, ovarian fibrothecomas usually appear as solid adnexal masses with delayed enhancement because of their fibrous composition [6]. Large lesions may show edema, cystic degeneration, hemorrhage, or heterogeneous enhancement, further complicating diagnosis [7].

### Importance of fertility preservation

The most important teaching point of this case relates to patient age and surgical strategy.

In young women, misinterpretation of Meigs syndrome as disseminated ovarian cancer may lead to unnecessarily aggressive surgery including hysterectomy, bilateral adnexectomy, radical peritonectomy, or extensive cytoreductive procedures with major reproductive consequences.

In the present case, careful intraoperative reassessment and the absence of obvious carcinomatosis allowed conservative unilateral adnexectomy with preservation of the contralateral adnexa and future fertility potential.

Awareness of this rare benign entity is therefore essential for both radiologists and surgeons. Recognition of imaging clues favoring fibrothecoma may help avoid overtreatment and guide fertility-sparing management whenever oncologically appropriate.

## Conclusion

Ovarian fibrothecoma associated with Meigs syndrome represents a rare but important mimic of advanced ovarian malignancy. The association of ascites, pleural effusion, elevated CA-125, and suspicious peritoneal nodules may falsely suggest peritoneal carcinomatosis, particularly in young women.

This case highlights the critical role of radiologic-pathologic correlation and cautious surgical decision-making. Increased awareness of this diagnostic pitfall may prevent unnecessarily aggressive surgery and preserve reproductive potential in young patients.

## Patient consent

The authors confirm that written informed consent was obtained from the patient for publication of this case report and any accompanying images, in accordance with the ethical standards of Radiology Case Reports (Elsevier).

## Appendix Supplementary materials

Video 1 Axial CT cine reconstruction demonstrating left pleural effusion, abundant intraperitoneal ascites, giant right ovarian mass, and normal appearance of the left adnexa. Image, video 1 dummy alt text

Video 2 Intraoperative video showing the giant right ovarian mass and preserved normal left adnexa before right adnexectomy. Image, video 2 dummy alt text

## References

[1] Young R.H. (2018). Ovarian sex cord-stromal tumours and their mimics. Pathology.

[2] Scully R.E. (1977). Ovarian tumors: a review. Am J Pathol.

[3] MEIGS J.V. (1954). Fibroma of the ovary with ascites and hydrothorax; Meigs' syndrome. Am J Obstet Gynecol.

[4] Riker D., Goba D. (2013). Ovarian mass, pleural effusion, and ascites: revisiting Meigs syndrome. J Bronchology Interv Pulmonol.

[5] Krenke R., Maskey-Warzechowska M., Korczynski P., Zielinska-Krawczyk M., Klimiuk J., Chazan R. (2015). Pleural effusion in Meigs' Syndrome-transudate or exudate?: systematic review of the literature. Medicine (Baltimore).

[6] Outwater E.K., Wagner B.J., Mannion C., McLarney J.K., Kim B. (1998). Sex cord-stromal and steroid cell tumors of the ovary. Radiographics.

[7] Jung S.E., Lee J.M., Rha S.E., Byun J.Y., Jung J.I., Hahn S.T. (2002). CT and MR imaging of ovarian tumors with emphasis on differential diagnosis. Radiographics.

[8] Toumi D., Mnejja A., Ghaddab I., Bergaoui H., Chaouch M.A., Zoukar O. (2024). Challenging diagnoses in a case report: ovarian fibrothecoma with elevated CA125 levels mimicking malignancy. Int J Surg Case Rep.

[9] Samanth K.K., Black W.C. (1970). Benign ovarian stromal tumors associated with free peritoneal fluid. Am J Obstet Gynecol.

[10] Timmerman D., Moerman P., Vergote I. (1995). Meigs' syndrome with elevated serum CA 125 levels: two case reports and review of the literature. Gynecol Oncol.

[11] Lin J.Y., Angel C., Sickel J.Z. (1992). Meigs syndrome with elevated serum CA 125. Obstet Gynecol.

